# The History of British Fossil Mammals and Birds

**Published:** 1846-04

**Authors:** 


The History of British Fossil Mammals, and Birds. By *R. Owen*, F.R.S., &c. Octavo, pp. 560. London, 1846. Van Voorst.

This masterly manual of Palæontological Anatomy must be in the hands of every one, who takes an interest in the history of the fossil remains of the higher vertebrate animals. The apocalyptic view which it presents of these tenants of the primæval world, ere man was formed to subdue and occupy the earth, suggests many a strange and startling reflection. Some rash and hasty observers in this field of enquiry have been led into the devious paths of a presumptuous and perplexing scepticism. But such will not be the case with the well-disciplined student of Nature. Each successive disclosure of her works—whether these be patent as the eye of day, or only seen " as through a glass, darkly"—will but impress his mind with a sense of deeper reverence before Him, by whom and for whom all things were created. It is not given to us to understand the end and design of many of the operations of His hand; but of this we may be assured, that the book of Creation, if looked at aright, will ever be found to harmonise with, and to throw light upon, that of Revelation.

It would be quite misplaced in a professional journal to review such a work as the present; but, as medical men of education are expected to be acquainted with the great and leading facts of geological Zoology, we strongly advise them all to peruse it. It contains elaborate descriptions of all the fossil relics of the higher vertebrates, from those " bones of contention" found in the Stonefield slate (one of the secondary groups of rocks), down to the remains that abound so largely in the diluvial soil of our country. Credit is due to the publisher for the admirable manner in which the work is *got up;* the paper and printing are excellent, and the woodcuts (237 in number) of unsurpassable beauty.

*Dr. Arnott on Therapeutic Enquiry*, pp. 57.—We regret that we cannot report favourably of this little work. Its design is good, and the subject is an interesting one; but then some of the statements which it contains are so strange, and one or two of its suggestions so extraordinary, that practical men will not be very willing to take Dr. A. as a guide in professional matters generally. Severe and continued vomiting is recommended as an almost infallible remedy in malignant sore throat. The inhalation of the nitrate of silver (suspended in the air) is proposed in diseases of the respiratory apparatus. The *modus operandi* of bloodletting, in removing rigidity of the uterus during parturition, is stated to be unknown. Allusion is made to the cure of a case of Hydrocephalus by the accidental evacuation of the fluid, from a nail penetrating the skull. In well-marked cases of apoplectic hæmorrhage (the usual seat of which is the *corpus striatum*), Dr. A. proposes that an opening be made through the skull, meninges, and the brain itself, for the removal of the effused blood! Need we comment upon this?

*On Scarlatina, &c. By J. B. Brown.* Pp. 66.—This is a very flimsy and offensive production—flimsy, because its story might have been told in a couple of pages; and offensive, because, under the guise of philanthropic and worthy professional motives, it is little better than a self-laudatory advertisement. Indeed, the author very coolly says that he wishes it to be read by parents and heads of families, (how disinterestedly considerate!) as well as by members of the medical profession." Distilled vinegar, in frequently-repeated doses of from half a tea-spoonful to a dessert spoonful, is declared to be an almost specific remedy for all forms of Scarlatina. We must not forget to add that Mr. B. applies lunar caustic—either solid, or dissolved in water (10 grains to the ounce) —to the throat, from the first blush of the disease. Such being this gentleman's *practice*, it matters little what may be his *theory*. Those, who wish to know, may consult his book.

*Dr. Monat on the Bengal Medical Returns.*—This is an ably-written pamphlet. Dr. M.'s strictures on the nosological tabular forms issued to the medical officers in the E. Indian army are, on the whole, perfectly just, and deserve the attentive consideration of those in authority.

